# Vaccination in the Light of the Royal British Commission

**Published:** 1897-05

**Authors:** M. R. Leverson

**Affiliations:** Fort Hamilton, L. I.


### VACCINATION IN THE LIGHT OF THE ROYAL BRITISH COMMISSION.

M. R. Leverson, M. D., Fort Hamilton, L. I.

(Continued from page 120.)

We left off with a narration of the exposure of Dr. Hopkirk's untruthfulness before the Royal British Commission with regard to the small-pox fatality in the Prussian and French armies during the war of 1870; we have now to describe his further conviction of falsehood before the same Commission, and especially with reference to the small-pox fatality in Prussia among children under ten years of age.

Having put in (Q. 1,523) a mutilated extract from Dr. Boing, purporting to be to the effect that there was no compulsory vaccination in Prussia prior to 1874—Dr. Collins, who seems to have been sufficient of a German scholar to realize that a fraud was being attempted, called upon him to continue his translation, which was: "I believe that the thorough vaccination of the population before the compulsory law of vaccination was promulgated was greater than at the present time." He also ran foul of Dr. Seaton, the procurer of the vaccination laws of England, and states that Dr. Seaton's allegation in 1871 that he knew Prussia to be well protected (Q. 6,794-5) was incorrect! At (Q. 1,469) Dr. Hopkirk states positively of the small-pox in Prussia: "None of the children under ten died who were vaccinated."

At (Q. 6,809) Dr. Collins put into his hand the Beiträge of the Berlin Sanitary Commission, and said (Q. 6,813): "Would you kindly look at page 168 of the same official work; that table gives the attack and also the deaths. Do you find there any cases of deaths among children under ten years of age from small-pox, who had been vaccinated or revaccinated? Ans. Certainly."

(Q. 6,814.) "Would you read out the numbers?" "In the first year (of life) there were 259 cases and 136 deaths; from one to six there were 1,244 cases and 437 deaths; from the sixth year to the tenth year there were 737 cases and 133 deaths." The vaccinationists on the Commission tried hard to help Dr. Hopkirk out, but the more they tried, the deeper he floundered, and so we will only now say, "Requiescat in pace!"

Before proceeding further in presenting to the readers of THE HOMEOPATHIC PHYSICIAN the testimony given before the Royal British Commission, it is well to call attention to the curious fact that although the testimony of four of the most prominent of the witnesses for vaccination has thus far been heard, not one of them has attempted to tell the Commission what vaccination, or the vaccine virus, or even vaccinia is!

Were the present review of that testimony published altogether it would be sufficient to call attention to that fact when it was "all in;" but obliged to present it in a serial form, I think it desirable to call attention to the fact now, and to ask the reader to bear it well in mind. Sir John Simon, when asked how to distinguish true cow-pox from the "spurious" (Q. 192), referred the Commission to "an expert," Mr. Thorne Thorne (Q. 801), was afraid his opinion as to the origin of vaccine lymph would be of very little interest to the Commission, as he had never studied the question.

Mr. Rauch, of Illinois, who disgraced the United States by his palpable ignorance and untruthfulness, while he boasted of the many despotic and unlawful acts he had committed in his character of Health Officer at Chicago, did not know anything about the "lymph" he used (Q. 1,110), though he believed it was of the Beaugency stock, but what it was, or how he knew it to be genuine, he could not tell.

We will now proceed to the testimony of Dr. Wm. Gayton, Superintendent of the Northwestern Fever Hospital for the past six years, prior to which he was medical superintendent of the Homerton Small-pox Hospital from 1870 to 1883. In 1881 he took a chief part in the organization, and had medical charge of a small-pox camp at Darenth,* and in 1882 was visiting medical officer of the hospital ships for small-pox (Q. 1,683).

Dr. Gayton's experience has extended to 12,920 cases, but excluding 2,104 cases of convalescents at the camp at Darenth, and 413 of mistaken diagnosis, the number of small-pox cases which he has personally treated or supervised amounts to 10,403 (Q. 1,685). He thinks (Q. 1,686) that if the system of vaccination and re-vaccination could be rigidly enforced, and the operation properly performed, it would prove, as Jenner anticipated, an all-powerful protection against small-pox.

Here is to be noted an important modification of the Jennerian nostrum. Not only was re-vaccination unnecessary according to Jenner, but it was impossible, and so recently as 1851 the Directors of the National Vaccine establishment declared "that the restriction of the protective power of vaccination to any age, or to any term of years is an hypothesis contradicted by experience, and wholly unsupported by analogy."—Report of the National Vaccine Establishment, 1851.

With great industry Dr. Gayton has prepared a table, which he handed in (Q. 1,687, Appendix III, Table A, pp. 243-4, to second report of the Commission), showing the vaccinal condition of these 10,403 cases, and of the fatality of the small-pox among them.

He presented in Table B, p. 245, a condensed analysis of the former table, which he thought showed that in the unvaccinated the fatality at each age period was greater as com-

* I have been unable to find this name in any of the numerous gazetteers I have consulted.

pared with the respective fatalities among those vaccinated with good, and those with imperfect marks, and those said to have been vaccinated, but with no marks visible; but under the questioning of Mr. Picton and of Dr. Collins his classification broke down. In (1,706) he says: "When I had one good mark and three imperfect marks I invariably ignored the three imperfect marks, and took the good mark." And (1,827), he tells the Commission that vaccination marks wear out with time. He also says (1,775), I think primary vaccination is a very fleeting protection, indeed. As to the time that primary protection lasts, I do not know, but I think it is a very short time, and (1,768) he says it is not absolutely protective up to any age whatever, because his table shows some cases under two years of age. It must be remembered that the pretext under which vaccination has been forced upon the people is that of Jenner, constantly repeated by his followers until a very recent period, that "what renders the cow-pox virus so extremely singular is, that the person who has been thus affected is forever after secure from the infection of small-pox." Dr. Gayton told the Commission (1,809) that of the 10,403 cases, 8,234 had been vaccinated, and (1,811) "that post-vaccinal small-pox is of common occurrence, and brings by far the larger proportion of inmates to the small-pox hospitals, practically eight-tenths of them." Also (1,812) that in some well-vaccinated subjects modification of small-pox is not observed. He has seen (1,814) hemorrhagic small-pox in well-vaccinated persons, and as this form is generally fatal, it would be difficult to see how vaccination "mitigated" the disease in these cases. He also admitted that he considered the fact of a person presenting himself at the end of the third day with confluent small-pox, to be *prima facie* evidence of the absence of vaccination! (1,821.) So that Dr. Gayton's statistics were compiled upon the principle, not that persons escaped small-pox, because they were vaccinated, but that they were unvaccinated because they had severe small-pox. The whole of the theory of "mitigation" is based upon statistics thus compiled.

Dr. Gayton has no knowledge as to the origin of the lymph used by the Government vaccinators (1,829), and notwithstanding his large experience, both of cow-pox and of small-pox, he has no opinion with regard to any relationship between them (1,834); neither is he able to give any opinion as to whether a person is better protected when re-vaccination has succeeded, or when it has failed (1,836). He admits (1,842), that the unvaccinated class are as a rule from a poorer class than the vaccinated, and (1,843) that this would operate to raise the unvaccinated mortality, but he does not think (1,844) that any unsanitary condition would start small-pox. It will perhaps not be out of place to notice here that while it is true that unsanitary conditions alone are not sufficient to start either small-pox, or any other epidemic, experience proves that it takes on an epidemic form only when that which the prince of observers, Sydenham, termed "An epidemic condition of the atmosphere" acts in conjunction with filth. This observation of the profound Sydenham has been confirmed by modern science. That which Sydenham termed an "epidemic constitution of the atmosphere" is now with a little more approach to exactness described as a peculiar electric condition—sometimes a deficiency of ozone—and one of the deplorable results of the Jennerian superstition is that attention has been generally diverted from researches tending to throw light on this important question. In (Q. 1,845) Dr. Gayton admits that overcrowding extends an epidemic of any kind, whether it is small-pox or fever. In (Q. 1,853) he admits that the hospital fatality of small-pox cases in pre-vaccination days was eighteen per cent. He is unable to explain how it is that eighty per cent. of the present small-pox cases are vaccinated, nor yet that while the total hospital small-pox fatality continues at eighteen per cent. as in pre-vaccination days, what he terms "natural small-pox" gives a mortality of forty per cent.

In spite of all the admissions thus made by this unquestionably honest witness, when Mr. Hutchinson says (1,865), I suppose you are an advocate for the repetition of vaccination? he answers: "Certainly."

The next witness was Dr. Barry, whose evidence turned chiefly on the Sheffield small-pox epidemic of 1888. The report made by this gentleman upon that epidemic seemed to present a solid advantage for vaccination. His cases, until subjected to really scientific examination, present an appearance of careful investigation and of incisive analysis. His testimony occupied nearly the whole of five sessions of the Commission, viz., the 16th, 18th, 23d, 25th, and 30th of October, 1889, and takes up fifty-three pages of their second report, besides in the appendix a large number of elaborate diagrams, together with a map of Sheffield and its environs. Alas! all this labor and ingenuity were absolutely wasted. Whether Dr. Barry believed in his own story can never be told. It is probable that he did, so powerful do the biases of education, profession, and self-interest warp the intellect and render it incapable of perceiving truth.

The unconscious bias of his mind becomes evident at once (1,883) when he states that being sent to Sheffield by the Local Government Board of England (a body having functions similar to our State and local Boards of Health), the only remedy which occurred to him was to secure the further vaccination and re-vaccination of the already frightened inhabitants of that much vaccinated city, vaccinated according to the official estimates up to ninety-eight per cent. of the population.

It is admitted even by vaccinationists that nothing is so calculated to spread any epidemic as scare. There is a well-known story ascribed to a witty French physician to the effect that in 1832 King Death met his Viceroy, Cholera, coming out of Paris. King Death commended his lieutenant for the noble services he had just performed, to which Viceroy Cholera replied: "Sire, I do not deserve your commendations, they belong to your majesty's Viceroy Fear. Where I killed one, Scare slew ten!"

Yet with this knowledge, common to every educated physician, Dr. Barry caused posters to be placed upon walls, and hand-bills to be distributed, and house-to-house visitations to be made to secure vaccination and re-vaccination (Q. 1,883).

Notwithstanding this perfect vaccination up to ninety-eight per cent of the population (as it was said), there were among her 316,000 inhabitants 6,088 cases and 590 deaths. Of 830 troops quartered there, every man of them re-vaccinated, men not only in the prime of life, but of selected vitality, twelve were attacked and one died. Of the 6,088 cases and 590 deaths, 5,035 cases and 246 deaths were admitted to have been well vaccinated; but as the old trick was resorted to of recording as unvaccinated nearly every case in which the marks could not be seen, and as in a large proportion of the vaccinated confluent cases, the confluence of the pocks would render vaccination scars absolutely invisible, the worthlessness of such statistics must be evident.

But further, whenever any child under ten years of age reported as vaccinated was attacked with small-pox Dr. Barry says: "I personally inspected the child," (1,896), "and if I found that it was unvaccinated the return was corrected." Now, it is evident that the most accurate mode of determining whether a child under ten years of age had been vaccinated would have been to refer to the official registers, for under the English system the official vaccinators are paid only for successful vaccinations. He admits (2,095) that when a boy is to be received into a school which requires proof of vaccination, the parents can get the proof of his vaccination from the vaccination registers, but when Mr. Picton said to him (2,096), "Then I should like to know why you did not make any attempt to compare the information of parents or neighbors with the vaccination registers?" he replied, "The whole of the figures are given here," and when further pressed (2,097), excuses his not having done so on the plea of the great length of time it would have taken to do so!

Notwithstanding the alleged ninety-eight per cent. of vaccination, the epidemic of 1887-88 is shown to be the next severest to that of 1871-72 of all the epidemics since 1863 (2,106-2,112).

(Q. 2,116 to 2,130) are directed to the bearings of the small-pox mortality upon the general death rate. (Q. 2,131) shows the small-pox mortality at its highest incidence, viz., 245 per 1,000 in 1871, while the general death rate fell nearly two per 1,000; and (2,134) Dr. Barry admits that it does not at all follow that a reduction in deaths from small-pox will reduce the general mortality. In (Q. 2,137-8) he tells us that from 1873 to 1887 the small-pox mortality was below the average in every year except the last, while the general mortality was above the average in six of the years 1873 to 1887; but he seeks to diminish the effect of this statement by saying that it is greatly below the average of the twelve preceding years. Now as nearly the greatest epidemic of the century occurred in 1871 and 1872, and the death rate of that epidemic year as seen in (2,131) was two per 1,000 below the average, the fact thus stated by Dr. Barry, for the purpose of diminishing the force of his admission in (2,137-8), given above, serves rather to accentuate the fact first observed by Dr. Robert Watt, Glasgow, and which Dr. Gregory termed "the law of vicarious vitality," and defined as being "that whenever one epidemic diminishes, another increases, so that the sum total of epidemic mortality remains on an average of years nearly the same." (Gregory on Eruptive Fevers, pp. 5-6.)

This law points so clearly to a common cause for these diseases, that, knowing as we now do for certain in the case of cholera, that a certain electrical condition of the atmosphere plus filth is essential to its prevalence as an epidemic, one marvels at the blindness which fails to search for analagous conditions as the cause of small-pox.

In (Q. 2,140-1) Dr. Barry admits that Sheffield was a well-vaccinated town, that at the time when the epidemic broke out less than five per cent. of the births were unaccounted for, while (Q. 2,142) Dr. Buchanan stated in the introduction to the Sheffield report, XV, that "on an inquiry made less than one per cent. of the children of school ages were found to be unvaccinated."

In (2,151) Dr. Barry admits that the administration of the law (for compulsory vaccination), so far as it was carried out in Sheffield did not prevent the epidemic there. In (Q. 2,154) he stated that the claim for vaccination as established by the facts of the Sheffield epidemic (supposed facts, we shall presently see that they were not facts) is limited to this, that the unvaccinated will take the disease in a larger proportion than the vaccinated, will generally have it worse, and will die in a greater proportion; also that they will spread the disease probably more than others (another bold assumption, for which neither the Sheffield statistics, nor any other give the slightest warrant). The experience of Sheffield, he said, shows that a larger proportion of the unvaccinated had small-pox of the worst type. (Q. 2,159 to 2,165) refer to observations of certain peculiarities of the season leading to one of the causes of the existence of greater filth than usual, but no one seems to have been concerned to search for that "epidemic constitution of the atmosphere," which the sagacity of Sydenham discerned more than 200 years ago, so deplorably have the minds of so-called men of science been diverted from the really important paths of research, which would lead them to the real conditions of vitality and morbidity. In (2,167 to 2,254) Dr. Barry is questioned as to the sanitary condition of the various parts of Sheffield, with the result that where this condition was worst, and the population most crowded, there were the greatest amount of small-pox and the worst cases, and that the opposite was true in the best sanitary parts of the town; and though he tried to soften these facts by various explanations, his own report, as read to him in (2,169) and (2,175), prove the facts too conclusively to be altogether denied (2,176 and 2,177).

Mr. Picton questions Dr. Barry on some of the cases: Case 10, Mary F., set down in Dr. Barry's return as unvaccinated, was stated by the woman who nursed her to have been vaccinated, as she (the nurse) stated she saw the marks upon her arm. (2,307), Case 11, Hannah P., was nursed by an unvaccinated sister, who did not take the small-pox. No. 16, nursed by a vaccinated mother, who took small-pox. Case No. 17, Allen L., put down as unvaccinated owing to ill-health, but reckoned among the unvaccinated, had consumption. Case No. 32, entered as unvaccinated, stated by the mother to have been vaccinated, and so forth, showing numerous errors of this kind. It should be stated that Dr. Barry professes to have no knowledge of the facts upon which he is questioned by Mr. Picton, but as the truth of those facts was established by other testimony at a later period, I have introduced them here for the sake of convenience.

In (2,370) the "scare" notice before mentioned is referred to, but in justice to Dr. Barry it should be stated that he said he had nothing to do with the drawing of it up. It was, however, admitted by him to have been issued as a result of his "personal conference with the local authorities." (Q. 2,372), and that it was seen by him (2,374), and that he never expressed the slightest disapproval of it (2,375); nevertheless it contained this palpable falsehood, contradicted by Dr. Barry's own statistics, viz.: "Out of three thousand cases of small-pox that occurred in Sheffield, not one single successfully revaccinated case has been reported as having died."

But the whole fabric ingeniously attempted to be constructed out of Dr. Barry's report was soon to be tumbled upon the heads of its inventors.

Making every conceivable deduction, Dr. Barry had been forced to the admission that 451 children under ten years of age who had been successfully vaccinated had suffered from small-pox (Q. 2,042).

This was attempted to be whittled away in his report as follows (*Second Report*, p. 49a):

UNDER TEN YEARS OF AGE.

| *Vaccinated.* | *Unvaccinated.* |
| --- | --- |
| Of 338 (*sic*, really 451 as a minimum) (Q. 2,042) vaccinated children in the total population of Sheffield attacked by small-pox 7, or 2.1 per cent., died. | Of 402 unvaccinated children in the total population of Sheffield attacked by small-pox 130, or 32.4 per cent., died. |

It is to be borne in mind that this number of unvaccinated includes children under three months, and those whose vaccination had been postponed because of sickness. Again on the same page:

AGED TEN YEARS AND UPWARDS.

| *Vaccinated.* | *Unvaccinated.* |
| --- | --- |
| Of 4,642 vaccinated persons in the total population of Sheffield attacked by small-pox 236, or 5.1 per cent., died. | Of 625 unvaccinated persons in the total population of Sheffield attacked by small-pox 190, or 30.4 per cent., died. |

The reader will remember (*supra*) the device by which the severe cases were thrown into the unvaccinated class.

Again on p. 39a we have:

| *Vaccinated.* | *Unvaccinated.* |
| --- | --- |
| Of the 268,397 persons of all ages returned as vaccinated 4,151, or 1.55 per cent., had been attacked by small-pox, and 200, or 0.07 per cent., died. | Of the 5,715 persons of all ages returned as unvaccinated, 552, or 9.7 per cent., had been attacked by small-pox, and 274, or 4.8 per cent., died. |

Over ninety comparisons of this sort were made in Dr. Barry's report, and, as stated above, did they represent the real facts, would show that a vaccinated person had less chance of taking small-pox than an unvaccinated one, and if attacked was less liable to have it severely, or to die.

To those who have not observed the conduct of the British Local Government Board, or of our own Boards of Health, what follows would almost seem incredible. It will, however, be proved.

This apparently comparative immunity of the vaccinated will be shown to have been a statistical trick, detected, however, by the great statistical skill of, I believe, Mr. George S. Gibbs and Mr. Alex. Wheeler, and it was brought out by Dr. Collins in his able examination of Dr. Barry himself. (Q. 2,377-8.) The accuracy of the ratios which appear in parallel columns throughout the report, would be dependent on the accuracy of the returns of population contained in the vaccination census. (Q. 2,379.) During the period of the epidemic, from March, 1887, to March, 1888, and especially in March, 1888, vaccination and revaccination were very extensively practiced in Sheffield, so that (2,385) during the period of the epidemic the vaccinated population was considerably recruited, and the unvaccinated population was considerably diminished.

(Q. 2,405.) The vaccination census was not completed till the third week in March, 1888, near the period of the termination of the epidemic—that is (2,407), out of 680 deaths 590 had taken place before Dr. Barry closed his inquiry, and (Q. 2,424) the number of unvaccinated persons was very considerably diminished in March, 1888, compared with what it was in March, 1887, and the vaccinated population had been very considerably recruited.

(Q. 2,432.) The unvaccinated population at the time of the census upon which was assessed the unvaccinated attacks and the unvaccinated deaths was very much smaller than obtained during the period of the epidemic, or, at any rate, at the time of its commencement, and (Q. 2,433) the vaccinated population upon whom was assessed the attack rate and the death rate in the vaccinated was proportionately increased during the same period.

As it may be difficult fully to appreciate the cunning of the trick thus exposed, I will borrow the illustration of it given by the editor of *The Vaccination Inquirer*, Vol. XII, August, 1890, p. 78:

"To illustrate the fallacy in a simple form, let us suppose a village of 1,000 inhabitants, whereof 900 are vaccinated and 100 are not. Suppose small-pox to break out and attack equally and indiscriminately ten per cent. of both classes. There would be ninety vaccinated and ten unvaccinated cases. Suppose this process to be spread over a period of several months, during which those of the unvaccinated who are not attacked are persuaded to get themselves vaccinated If an investigation is now made of that village to determine the incidence of small-pox, it will stand thus:

| | Population. | Cases. | Percentage. |
|---|---|---|---|
| Vaccinated ...... | 990 | 90 | 9.09 |
| Unvaccinated .... | 10 | 10 | 100 |

Absurd as this conclusion is, it is only an extreme case of Dr. Barry's fallacy.

After this, a slight error in the census, such as the omission of some 40,000 of the population, becomes hardly worth noticing, except to emphasize the worthlessness of the testimony given or collected by officials who have an interest in upholding "power, place, and pelf." What an illustration of the truth of Bentham's analysis referred to above! (Vol. XVI, p. 497).

Dr. Barry's report gives us the fact (Q. 2,602) that of five children in one family, three, aged respectively eleven, fifteen, and sixteen, who had been vaccinated in infancy, all suffered from small-pox, and that the last two were badly pitted; that two other persons, aged fourteen and twenty respectively, who had never been vaccinated, and who slept with the others, did not contract small-pox!

Some facts of importance as having a bearing on the pretended auto-protection of small-pox, a doctrine older than that of vaccination, but, like it, adopted without a tittle of evidence in its support, appears at (Q. 2,040), where we are told that twenty-three persons had small-pox for the second time, thirteen of whom had also been vaccinated.

